# Impact of APOE4 Genotype and Lifestyle Factors on Alzheimer’s Disease Risk in Patients with Transient Ischemic Attacks: A Comparative Cohort Study

**DOI:** 10.1002/alz70861_108985

**Published:** 2025-12-23

**Authors:** Arwa Ayman Nagah Ahmed Shahin, Mohamed Khaled Ali Mohamed Ali Zid, Mohamed Hosni Kamel Elkellawi

**Affiliations:** ^1^ Cairo University Faculty of Medicine, Cairo, Cairo Egypt

## Abstract

**Background:**

Alzheimer's disease (AD) is a neurodegenerative disorder which affects millions of individuals worldwide and has numerous recognized genetic and environmental risk factors. The apolipoprotein E (APOE) gene and the APOE4 allele are one of the principal genes involved in genetic risk for AD. Transient ischemic attacks (TIAs) are short‐lived episodes of neurological impairment often considered predictors for stroke and possibly linked to having a higher risk of developing AD. This study explores the impact of the APOE4 genotype and lifestyle factors (such as diet, physical activity, and smoking) on the risk of Alzheimer’s disease in patients who have experienced TIAs.

**Method:**

This was a comparative cohort study conducted over a five‐year period. A total of 300 participants who had experienced a TIA were recruited, with 150 having the APOE4 allele and 150 without. Lifestyle interventions were quantified with standard questionnaires regarding diet, activity, and smoking status. Participants' cognitive loss was monitored through neuropsychological tests and neuroimaging methods like MRI for tracking structural change in the brain. The principal outcome was development of clinical Alzheimer's disease based on DSM‐V criteria.

**Result:**

85 participants out of a total of 300 developed Alzheimer's disease within the study period. APOE4 carriers were at much higher risk of developing AD, having an incidence of 45% as opposed to 25% in non‐carriers (p < 0.05). In carriers with adverse lifestyle determinants (sedentary habits, unhealthy diet, and smoking), the risk was even higher, with 60% incidence of AD. In contrast, those with healthier lifestyle (e.g., regular physical exercise, good diet) were at lower risk (35%) of developing AD, regardless of APOE4 status.

**Conclusion:**

APOE4 genotype is a significant genetic risk factor for Alzheimer's disease in TIA patients. Lifestyle is also a strong modifier of AD risk, with healthier lifestyle choices possibly reducing the risk in high‐risk subjects. These findings highlight the need for early lifestyle intervention in TIA patients, especially with the APOE4 allele, in an effort to postpone or prevent Alzheimer's disease.

